# Leaf trichomes affect caterpillar feeding in an instar-specific manner

**DOI:** 10.1080/19420889.2018.1486653

**Published:** 2018-08-09

**Authors:** Rupesh R Kariyat, Sean B Hardison, Aisling B Ryan, Andrew G Stephenson, Consuelo M De Moraes, Mark C Mescher

**Affiliations:** a Department of Biology, University of Texas Rio Grande Valley, Edinburg, TX, USA; b Integrated Statistics, Woods Hole, MA, USA; c Department of Biology, The Pennsylvania State University, University Park, PA, USA; d Department of Environmental Systems Science, ETH Zürich, Zürich, Switzerland

**Keywords:** Glandular trichomes, larval instar, *Manduca sexta*, non-glandular trichomes, *Solanaceae*

## Abstract

Leaf trichomes play well-established roles in defense against insect herbivores, both as a physical barrier that impedes herbivore movement and by mediating chemical defenses. However, little work has examined how different trichome types influence herbivory by herbivores at different stages of development. We examined whether caterpillar instar and trichome type (glandular or non-glandular) affected the ability of the specialist herbivore caterpillar *Manduca sexta* to initiate feeding on 11 Solanaceous species exhibiting variation in the density and type of leaf trichomes. Our results suggest that non-glandular trichomes are far more effective than glandular trichomes in deterring the initiation of feeding by first- and second-instar caterpillars. Meanwhile, neither glandular nor non-glandular trichomes significantly affected the ability of third-instar caterpillars to commence feeding. These findings suggest that while non-glandular trichomes deter feeding initiation by early instar caterpillars, the contribution of both trichomes on later instars may depend on effects after feeding initiation.

## Introduction

A great deal of research on plant resistance to herbivores has focused on the induction of defenses post herbivore feeding [1]. However, various structural defenses, including the waxy plant cuticle, leaf and internode spines, and trichomes, can act to deter herbivory even prior to the onset of feeding [2,3]. Trichomes are commonly considered a first line of defense [4–6] and come in two distinct forms: glandular and non-glandular. Glandular trichomes act in defense by producing toxic secondary metabolites to impede or kill herbivores and/or by serving as triggers for the activation of jasmonic acid (JA) mediated defenses [7,8]. In *Solanum lycopersicum*, for example, herbivore movement ruptures glandular trichomes and elicits the expression of JA-mediated defense genes even prior to leaf damage [7]. In the *Nicotiana tabacum-Manduca sexta* system, glandular trichomes have also been shown to release volatiles following caterpillar damage that are attractive to predators [9]. In contrast, non-glandular trichomes are thought to act primarily as mechanical barriers that restrict herbivore movement and thereby prevent feeding damage [3,10]. Although various defensive functions of both glandular and non-glandular trichomes have been characterized, their relative efficacy in deterring feeding by different herbivores or herbivores at different stages of development are not well understood.

Non-glandular trichome growth has been shown to be inducible through caterpillar herbivory, hinting at a more specialized role in direct defense compared to glandular trichomes. For example, we previously documented that *M. sexta* feeding on older leaves of *Solanum carolinense* induced a higher density of stellate trichomes (branched non-glandular trichomes with spikes) on both surfaces of new leaves after 21 days [5]. In the same system, we also demonstrated that non-glandular stellate trichomes cause physical injury to caterpillars and impede the ability of early instar caterpillars to locate the epidermis and commence feeding [3]. For neonate caterpillars of species like *M. sexta*, any delay in initiating feeding could potentially prove fatal due to increased risk of desiccation, starvation, and/or predation [11]. Later instars are thought to be less sensitive to leaf defenses and leaf nutritional quality [12], and we have observed that late-instar caterpillars tend to ingest trichomes along with leaf tissues while feeding. However, previous work has not explicitly tested how trichome composition affects the ability of neonate caterpillars to initiate feeding.

**Figure 1.**
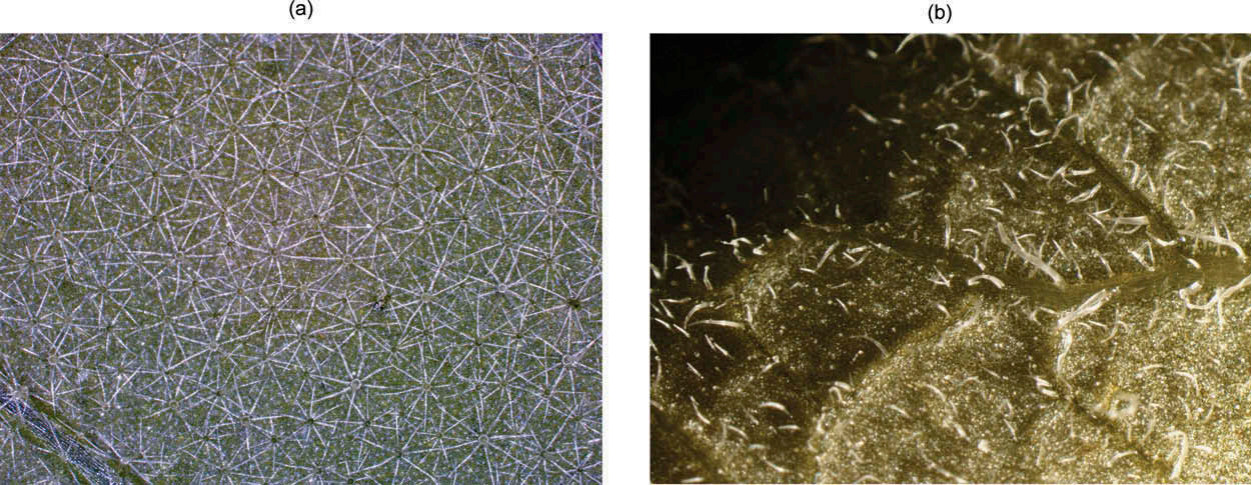
(a) Trichome morphology of *S. eleaegnifolium. S. eleaegnifolium* produce non-glandular stellate trichomes. Adaxial leaf surface magnified at 12X. (b) Trichome morphology of *S. tuberosum. S. tuberosum* produce glandular trichomes. Adaxial leaf surface magnified at 12X.

*Solanaceae* species are ideal for examining trichome based defenses as they exhibit considerable variation in trichome type and morphology. For example, two commonly cultivated species, *Solanum lycopersicon* (tomato) and *Nicotiana tabacum* (tobacco), have multiple types of glandular trichomes [7,9,13], whereas the common non-domesticated weeds *Solanum elaeagnifolium* and *Solanum carolinense* are devoid of any glandular trichomes, but have a dense mat of non-glandular stellate trichomes [3,5,14], (Figure 1). Here using multiple species from Solanaceae, we examined the effects of trichome type X *M. sexta* larval instar on feeding initiation.

## Results

### Non-glandular trichomes delay the onset of feeding more effectively than glandular trichomes

**Figure 2.**
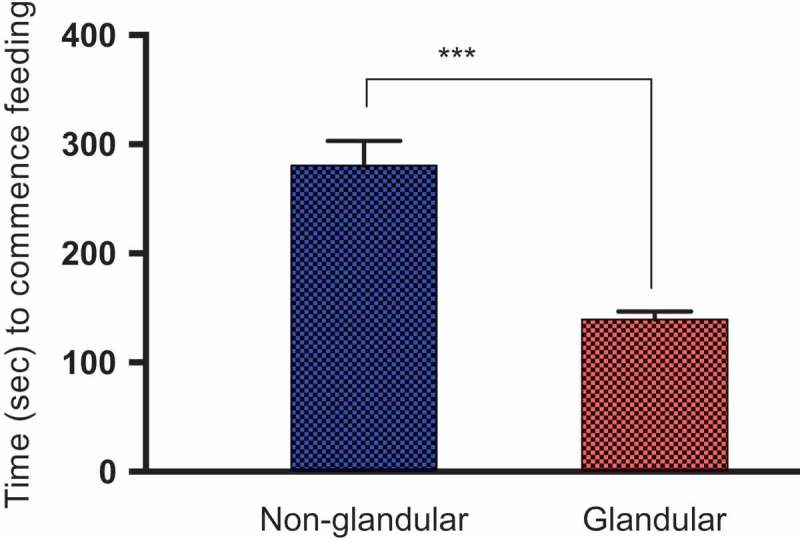
Time (in seconds) taken by *M. sexta* caterpillars to commence feeding on 11 Solanaceae species that vary in their trichome type (glandular or non-glandular). Asterisks (*) denote statistical significance at *P < 0.05*, ** P < 0.01, *** P < 0.001, and **** P < 0.0001.

**Figure 3.**
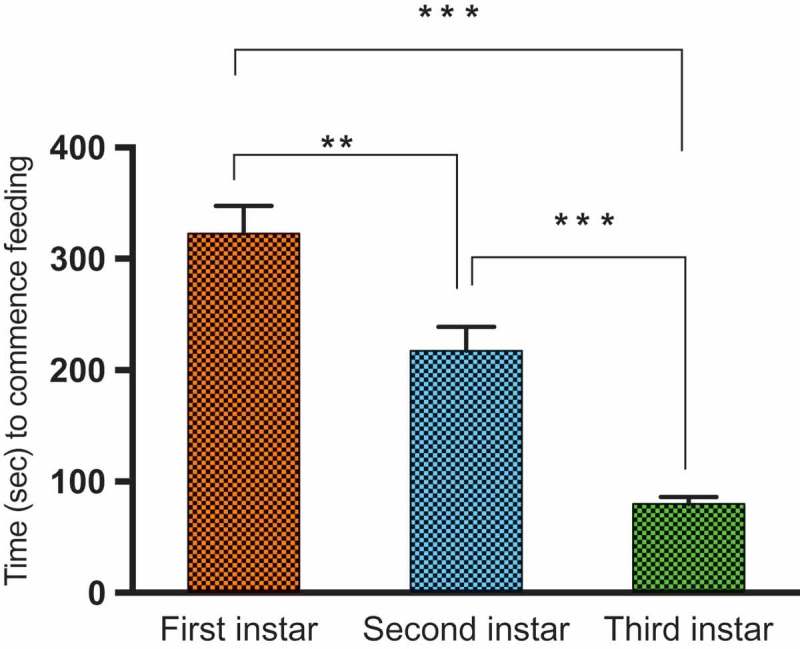
Time (in seconds) taken by first-, second-, and third-instar *M. sexta* caterpillars to commence feeding on 11 Solanaceae species. Asterisks (*) denote statistical significance at *P < 0.05*, ** P < 0.01, *** P < 0.001, and **** P < 0.0001. Results from pairwise post hoc Tukey comparisons.

**Figure 4.**
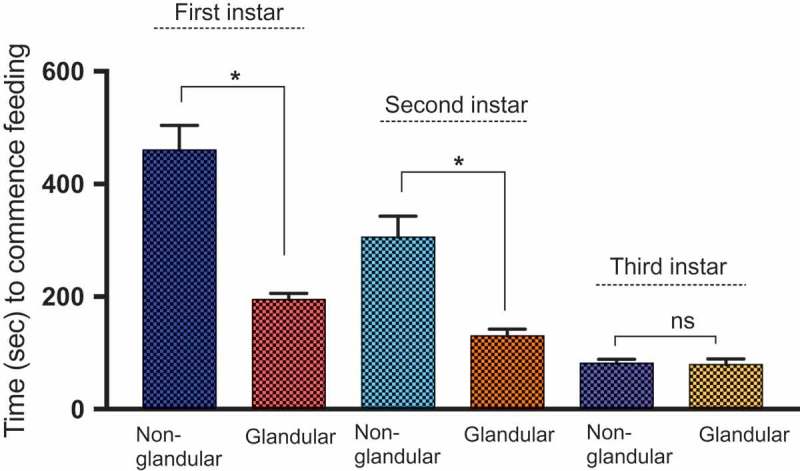
Pairwise comparisons of the effects of Instar X trichome-type interactions on time taken by first-, second-, and third-instar *M. sexta* caterpillars to commence feeding on 11 Solanaceae species varying in trichome type (glandular or non-glandular). Asterisks (*) denote statistical significance at *P < 0.05*, ** P < 0.01, *** P < 0.001, and **** P < 0.0001. Results from pairwise post hoc Tukey comparisons.

In this study, we compared the ability of *M. sexta* caterpillars of different instars to initiate feeding on Solanaceous species exhibiting either non-glandular or glandular trichomes. We found that regardless of caterpillar instar, non-glandular trichomes delayed feeding (by more than two-fold) when compared to glandular trichomes (Mean time to initial feeding 280.4 ± 22.7 vs. 139.5 ± 7.4 s respectively; Anova; F = 20.27, P < 0.00; Table 1, Figure 2; Supplemental video 1). First-instar caterpillars were also more effectively deterred by trichomes than later instars; For both glandular and non-glandular trichomes, first-instar caterpillars took longest to begin feeding (322.7 ± 24.77; Means ± SE; Two-way Anova; F = 92.34, P < 0.01 Table 1; Figure 3), followed by second (217.7 ± 21.15; Means ± SE) and then third instars (79.92 ± 6.113; Means ± SE). Pairwise comparisons also revealed that all instars differed significantly from one another. First-instar caterpillars on the three plant species that exhibit dense non-glandular stellate trichomes (*S. carolinense, S. eleaegnifolium*, and *S. melongena*) also took longer time to initiate feeding compared to those on *S. atropurpureum* and *S. aethiopicum*, which produce fewer non-glandular trichomes. For species exhibiting glandular trichomes, first-instar caterpillars also took significantly longer than second and third instars to commence feeding (Kruskal Wallis non-parametric One Way Anova; Kruskal Wallis statistic = 86.44; P < 0.0001). Pairwise analyzes of trichome type X larval-instar interactions revealed that first- and second-instar caterpillars took significantly longer to start feeding on species with non-glandular vs glandular trichomes (Two-Way Anova; Figure 4; P = 0.02, and 0.01 respectively; Table 1, Tukey post hoc tests). However, difference in trichome type made no difference for third-instar caterpillars, which were able to initiate feeding immediately after being placed on the leaf surface (P = 0.72). Interestingly, third-instar and later caterpillars also ingested trichomes when feeding (Supp. Video 1), unlike the early instars.

**Table 1.** Two-way ANOVA of time (in seconds) taken by first-, second- and third-instar *M. sexta* caterpillars to commence feeding on 11 *Solanaceae* species. ANOVA was carried out on log transformed data to meet normality assumptions.

Source	DF	MS	F	P
Larval instar	2	14.37	92.34	**0.000**
Trichome type	1	3.15	20.27	**0.000**
Larval instar X Trichome type	2	0.43	1.39	0.249
Error	366	0.15

## Discussion

We compared caterpillar feeding on 11 species of Solanaceae exhibiting variation in trichome type and density and found that non-glandular trichomes function more effectively than glandular trichomes as physical barriers to feeding. Both types of trichomes delayed feeding by early instar caterpillars. However, we found that non-glandular trichomes had little effect on the initiation of feeding in third-instar caterpillars. This result provides context for our previous report that third-instar caterpillars, while not deterred from initiating feeding, are detrimentally affected by non-glandular trichomes, which damage the peritrophic matrix [3]. Szyndler *et al*. [15] previously showed that the hooked trichomes in kidney bean (*Phaseolus vulgaris*) physically entrap and impale bedbugs (*Cimex lectularius*), thereby preventing them from moving on the leaf surface. We also observed physical damage to caterpillars (leakage of hemolymph) in our experiments, mainly caused by the sharp spikes of stellate trichomes.

The plant species used in our study included both cultivated crops and non-domesticated weeds. For example, *S. melogena* is a cultivated species and *S. eleaegnifolium* is an invasive weed; however, both maintain very similar non-glandular trichomes that successfully delayed early instar feeding. In species with dense stellate non-glandular trichomes (*S. carolinense, S. eleaegnifolium, and S. melongena*), early instar caterpillars often struggled to access the epidermis and were occasionally impaled by trichomes while navigating through the dense trichome mat (Supplemental video 1).

The greater delay in feeding onset observed for species with non-glandular trichomes is likely to be of particular importance for herbivore species like *M. sexta*, where the neonates are colorless and depend on ingested plant material to camouflage their bodies and reduce the risk of predation[11]. As demonstrated in previous studies [7,16], the mode of action of glandular trichomes differs from that of non-glandular trichomes as damage to glandular trichomes activates JA (Jasmonic acid) mediated defense gene expression and often contain toxins, resins, or signaling molecules rather than causing physical damage to caterpillars. The current results shows that glandular trichomes are less effective than non-glandular trichomes in preventing caterpillar herbivory for early instars. Both glandular and non-glandular trichomes weren’t effective in deterring feeding by late-instar caterpillars, suggesting that trichomes may also play a role in non- defensive functions such as water retention, temperature regulation, gas exchange, and UV protection [6,17].

Consistent with previous observations, we observed that later-instar caterpillars tend to ingest the stellate trichomes when feeding on leaves. We recently documented post-ingestion effects of such trichomes on caterpillars, reporting the gut peritrophic matrix was punctured and ripped by trichomes passing through the gut. Given the importance of the peritrophic matrix to nutrient uptake and protection against pathogens [18], it is not surprising that larvae also grew more slowly when feeding on plants with more trichomes [19] or on artificial diet containing trichomes compared to control diet [3].

In overview, the results of this study indicate the initiation of feeding on Solanaceous plant species by early instar caterpillars is strongly delayed by non-glandular trichomes but not by glandular trichomes, suggesting that the former play a primary role as a in deterring the initial inset of herbivory. Meanwhile, neither glandular nor non-glandular trichomes were ineffective in preventing third-instar caterpillars from initiating feeding, although each may mediate aspects of defense that come into play after the onset of feeding Future work will address the indirect immunological effects of trichome ingestion in third instar caterpillars [20], as well as examine the relative physiological costs of non-glandular trichome ingestion.

## Materials and methods

### Non-glandular Solanaceous species

The group of Solanaceous plants with non-glandular trichomes was composed of five species. Common horsenettle (*Solanum carolinense*) is a perennial shrub considered a noxious weed in its native range of eastern North America [3,5]. Multiple genets of *S. carolinense* plants used in this study were derived from the outcrossed fruits of 3–5 parental plants taken from a natural population near State College, PA, USA [21]. Seedlings of *S. carolinense* were transplanted to 15 cm pots after 3–4 weeks, and allowed to grow for 3–4 more weeks in a growth chamber (16: 8 Light and dark cycle, 23C, and 50% relative humidity) before used in experiments (For more details, see [3,5,21]).

The second non-glandular trichome producing Solanaceous species was silver leaf nightshade (*S. eleaegnifolium*) [14], a herbaceous perennial native to southwestern United States [22], which has become highly invasive in countries including Morocco, Australia, and Greece [23,24]. Plants used in this study were derived from populations in mainland Thessaloniki and the island of Lesvos, Greece. Collected rhizomes were allowed to resprout in a growth chamber (16: 8 Light and dark cycle, 23C, and 50% relative humidity) at ETH Zurich (Zurich, Switzerland), transplanted to 15 cm pots after 3–4 weeks, and allowed to grow for 3–4 more weeks before used in experiments. The third species in the non-glandular group was Aubergine (*Solanum melongena*). *S. melongena* plants used in this study were derived from the commercially available cultivar Nadia (Victory Seed Company, Oregon, USA). 10 days after germination, *S. melongena* seedlings were transplanted from flat beds to 15 cm pots. These were also and allowed to grow for 3–4 more weeks before being used in experiments under similar growth chamber conditions.

The fourth and fifth species in the non-glandular group were purple devil (*Solanum atropurpureum*) and aethiopian nightshade (*Solanum aethiopicum*). *S. atropurpureum* is a short-lived perennial shrub native to the state of Rio Grande do Sul, Brazil [25], and *S. aethiopicum* is native to west Africa; introduced into South America. Both *S. atropurpureum* and *S. aethiopicum* plants used in this study were derived from seed lots donated by Zurich Botanical Garden (Zurich, Switzerland). Both species were transplanted into 15 cm pots three weeks after germinating, and these were also allowed to grow for 3–4 more weeks before using in experiments under similar growth chamber conditions. Light microscopy (Leica M420 microscope with a camera attachment (Leica MC 170 HD) at magnification between 25 and 32 (Leica systems, Germany) showed that both *S. atropurpureum* and *S. aethiopicum* plants maintained non-glandular trichomes in lower densities when compared to *S. carolinense, S. eleaegnifolium*, and *S. melongena*.

### Glandular Solanaceous species

The species with glandular trichomes used in this study were tomato (*Solanum lycopersicon*), tomatillo (*Physalis philadelphica*), potato (*Solanum tuberosum*), Mapacho (*Nicotiana rustica*), Cape gooseberry (*Physalis peruviana*) and cultivated tobacco (*Nicotaina tabacum*). Seeds from commercially available store-bought varieties were used for all plants, with the exception of *S. tuberosum*, which were propagated using methods described for *S. melongena*. The Beef Steak variety of *S. lycopersicon* and Fiesta Duo variety of *P. philadelphica* were used from seeds bought from Renee’s Garden (California, USA) (Figure 1(D)). Seeds for *N. rustica* and *P. peruviana* were donated by Zurich Botanical Gardens (Zurich, Switzerland). *N. tabacum* seeds of the Dixie Shade variety were acquired from the Victory Seed Company (Oregon, USA). For *S. tuberosum*, a bag of the cultivar “Carola” was purchased and individuals cut into 10 cm tubers. Ten-day old *S. tuberosum* sprouts were then transplanted into 15 cm pots for 6–8 weeks before use in experiments.

For both glandular and non-glandular trichome groups, a preliminary experiment was carried out to ensure that *M. sexta* caterpillars are able to feed, grow, and pupate successfully after feeding each of the species used in the study.

### Rearing of M. sexta

*M. sexta* eggs were acquired from a lab colony maintained by the Max Plank Center for Chemical Ecology (Jena, Germany). Eggs received from this colony were hatched in a growth chamber (Relative humidity 60%, Temperature 24C, 16:8 day night cycle), and were were allowed to grow and develop on a wheat germ based artificial diet (Carolina biological Supply Company, NC, USA), and were then used in the assays.

### Bioassay

Individual first instar caterpillars (starved for ~ 4 hours) were placed onto fully developed young leaves of each of the 11 Solanaceous species (N = 12 −15 per species). For ease of data collection, only one caterpillar and plant were used at a time. After placing the caterpillar on the adaxial leaf surface, a stopwatch was used to record the time between placement of the caterpillar and the initiation of feeding. A similar approach was also used for second- and third-instar caterpillars, which were starved for 8–12 hours after molting in order to clear their gut contents.

### Statistics

To determine whether caterpillar instar and trichome type (i.e. glandular or non-glandular) affected caterpillar herbivory, we ran a two-way analysis of variance with time to initial feeding as the response variable, and trichome type, larval instar, and their interaction as predictors (General Linear Model). We also ran a non-parametric one way Anova (Kruskal-Wallis test for the effects of glandular trichomes on different instars. Anova’s were followed by Tukey’s HSD post hoc tests to examine pairwise comparisons between factors of interest. All analyzes and plotting were carried out using the statistical softwares Minitab 16 (PA, USA) and Graph Pad prism (CA, USA).
